# Validation of a Spanish Questionnaire on Mobile Phone Abuse

**DOI:** 10.3389/fpsyg.2018.00621

**Published:** 2018-04-30

**Authors:** María A. Olivencia-Carrión, Isabel Ramírez-Uclés, Pablo Holgado-Tello, Francisca López-Torrecillas

**Affiliations:** ^1^Center for Research into the Mind, Brain and Behavior, Granada University, Granada, Spain; ^2^Department of Personality, Assessment and Psychological Treatment, Universidad Nacional de Educación a Distancia, Madrid, Spain; ^3^Department of Behavioral Sciences Methodology, Universidad Nacional de Educación a Distancia, Madrid, Spain

**Keywords:** mobile phone, DSM-5, validity, Spanish population, abuse

## Abstract

Mobile phone addiction has attracted much attention recently and is showing similarity to other substance use disorders. Because no studies on mobile phone addiction had yet been conducted in Spain, we developed and validated a questionnaire (Cuestionario de Abuso del Teléfono Móvil, ATeMo) to measure mobile phone abuse among young adults in Spanish. The ATeMo questionnaire was designed based on relevant DSM-5 diagnostic criteria and included craving as a diagnostic symptom. Using stratified sampling, the ATeMo questionnaire was administered to 856 students (mean age 21, 62% women). The MULTICAGE questionnaire was administered to assess history of drug abuse and addiction. Using confirmatory factor analysis, we found evidence for the construct validity of the following factors: Craving, Loss of Control, Negative Life Consequences, and Withdrawal Syndrome, and their association with a second order factor related to mobile phone abuse. The four ATeMO factors were also associated with alcoholism, internet use, and compulsive buying. Important gender differences were found that should be considered when studying mobile phone addictions. The ATeMo is a valid and reliable instrument that can be used in further research on mobile phone abuse.

## Introduction

The mobile phone has many characteristics that make it attractive to young adults. It is primarily used to communicate but also has many other interesting applications, including camera, internet, music reproduction, games, and social media. The International Telecommunication Union report (The International Telecommunication Union, 2016) finds that 98% of young adults own a mobile phone in Europe and other studies indicate that young women in particular have more interest in mobile phones than other groups do (Roberts et al., 2014). There is evidence that mobile phone abuse in related to physical and mental wellbeing problems, including social and psychological disturbances such as attention deficit and hyperactivity disorder, disruptive behavior disorders, anxiety disorders, mood disorders, substance use disorders, sleep disorders, and eating disorders (Billieux et al., 2014; Foerster et al., 2015). In recent years, a co-occurrence has been established between mobile phone dependence and other behavioral disorders such as internet addiction (Chiu et al., 2013), compulsive buying (Jiang and Shi, 2016) and alcohol use (De-Sola et al., 2017a) or use of other substances (Gallimberti et al., 2016). However, it remains unclear if an individual that develops one addictive behavior (i.e., mobile phone abuse) is more likely to develop another addictive behavior or a substance use problem.

Although a definition of mobile phone abuse has not yet been agreed upon, some researchers define mobile phone dependence as a constant use of the device with a poor capacity to control daily activities, exhibiting extreme nervousness and aggressive behavior when deprived of its use; this excessive use is also accompanied by a progressive deterioration in school/work performance and social and family functioning (Billieux et al., 2014; Lin et al., 2015). These symptoms have a major negative impact on the life of the affected person, reflected in impaired health or deprived social functioning; they have also been shown to be equivalent to substance dependence as understood by the current nosological systems Diagnostic and Statistical Manual of Mental Disorders, Fifth Edition (DSM-5, American Psychiatric Association, 2012).

Mobile phone addiction could in many ways be similar to substance dependence disorders (Foerster et al., 2015; Roser et al., 2016). For instance, the abuse of psychotropic drugs (heroin, cocaine, cannabis, etc.) and alcohol is a complex social, biological, and psychological phenomenon. Whether an individual ever uses alcohol or another substance, and whether that initial use progresses to a substance use disorder of any severity, depends on a number of factors. These include: a person's genetic makeup and other individual biological factors; psychological factors related to a person's unique history and personality; and environmental factors, such as the availability of drugs, family and peer dynamics, coping with stress, and access to social support. Chronic consumption of several drugs (cannabis, stimulants, and opioids) has been associated with the presence of neuropsychological impairments in a broad range of functions. In recent years neuropsychological research on substance abuse has focused on the study of impairments in executive functions linked to the prefrontal cortex and their influence on the personality, cognitions, and behaviors of the substance abusers (López-Torrecillas et al., 2000; Verdejo-García et al., 2004).

To date, pathological gambling is the non-substance related addiction which has received most attention and has been examined extensively. The results reveal a number of substantial similarities between pathological gambling and substance-related addictions concerning phenomenology, epidemiology, personality factors, genetics, neurobiological processes, recovery, and treatment (Walther et al., 2012; Contreras-Rodríguez et al., 2016; Navas et al., 2017). In DSM-5, pathological gambling is classified as a non-substance-related addiction and is, therefore, removed from the former category “Impulse-Control Disorders” and included in the new “Substance Use and Addictive Disorders” category. Other potential non-substance-related addictions are internet addiction, compulsive buying, sex addiction, and mobile phone addiction, although these are not yet officially defined as disorders due to a lack of evidence. Despite a substantial overlap, it is not yet clear why some people become vulnerable to these behaviors. The co-occurrence of non-substance-related addiction with different forms of substance abuse such as smoking, drinking, use of cannabis, and other illegal drugs among young people has been repeatedly discussed (Vanyukov et al., 2012; De-Sola et al., 2017a).

The literature also reveals an association between multiple substance use and other risk behaviors among young adults. For example, binge drinking, cannabis use, and tobacco use appear to be more prevalent in young people (Van Rooij et al., 2014; Abebe et al., 2015). The use of both alcohol and cannabis predicts use of common addictive substances (Osuch et al., 2013; Viola et al., 2014; Vorspan et al., 2015) and tends to be accompanied by gambling (Larsen et al., 2013; Míguez and Becoña, 2015). In addition, a number of authors (Mudry et al., 2011; Yau et al., 2012; Grant et al., 2013; Lee et al., 2013; Mattebo et al., 2013; Schuster et al., 2013; Van Rooij et al., 2014; Biolcati, 2015) have pointed out the relationship between the amount of time young adults spend gambling, abusing their mobile phones, using the internet, playing video games, buying compulsively, or having sex and increases in alcohol, tobacco, cannabis, and drug consumption.

The acknowledgement of behavioral addictions as disorders can be traced as far back as Marlatt et al. (1988), who reported a repetitive habit pattern that increases the risk of disease and/or associated personal and social problems. Addictive behaviors are often experienced subjectively as a loss of control in which the behavior continues to occur despite volitional attempts to abstain or moderate use. Furthermore, in the last decade, a growing number of studies have established psychological and neurobiological similarities between the excessive practice of addictive behaviors (e.g., mobile phone abuse, compulsive buying, sex, internet, video gaming, and eating disorders; Billieux et al., 2010; Mentzoni et al., 2011). Research on the neurobiology of addiction has also found a common mechanism between substance addictions and behavioral addictions (Leeman and Potenza, 2013; Weinstein and Lejoyeux, 2015). However, at this point we do not know whether having one addictive behavior increases the likelihood of developing other addictive behaviors or other dependencies such as substance use disorders. In addition, alcohol, drugs, and pathological gambling may not be the only crippling addictions that we should address. Unfortunately, other addiction statistics are scarce because many destructive habits are not yet officially recognized as addictions, including mobile phone addiction, game addiction, eating, shopping, and sex addiction, all of which are problematic for many reasons. They all involve direct manipulation of pleasure through the use of products, similar to drug use disorders and food-related disorders.

The concept of non-substance-related (or “behavioral”) addiction describes syndromes analogous to substance addiction, but with a focus on a certain behavior which, similar to substance consumption, produces short-term reward and may persist despite harmful consequences due to diminished control over the behavior. Given that addictive behavior is not necessarily restricted to substance consumption, the DSM-5 broadens the category “Substance-Related Disorders” to a “Substance Use and Addictive Disorders” category including both substance and non-substance-related addictions. The Diagnostic and Statistical Manual of Mental Disorders—4th Edition (DSM-IV; American Psychiatric Association, 2002) conceptualized two discrete substance use disorders (SUD), abuse and dependence, defined by mutually exclusive sets of diagnostic criteria. Abuse required endorsement of one or more (1+) of four abuse criteria, and dependence required endorsement of three or more (3+) of seven dependence criteria. In contrast, the proposed Diagnostic and Statistical Manual of Mental Disorders—5th Edition (DSM-5; American Psychiatric Association, 2012) conceptualizes a unitary SUD construct, varying only in terms of severity. The literature reviewed here includes studies on postulated behavioral addictions related to the use of mobile phones, shopping, sex, internet, video gaming, and food, along with other studies that analyzed the co-occurrence of these addictions with substance abuse (for instance tobacco, alcohol, and cannabis substances). However, these are not included in the DSM-5 because of current lack of evidence. In order to be able to obtain relevant evidence in the first place, we need valid and reliable instruments that allow us to measure addictive behaviors such as mobile phone abuse.

The study of mobile phone abuse started in 2004 with the development of the Mobile Phone Dependency Questionnaire (CPDQ; Toda et al., 2004) designed for use in university populations and validated in a population of high school students by Kawasaki et al. (2006). Another instrument available for use in adult populations is the Mobile Phone Problem Use Scale (MPPUS; Bianchi and Phillips, 2005); including a recent short version (Foerster et al., 2015) and a version for teenagers [Mobile Phone Addiction Scale (MPAS; Leung, 2008)]. This scale has been translated into Japanese (Takao et al., 2009) and Spanish (López-Fernández et al., 2012), with some items previously translated for use in the Spanish university population (Ruiz-Olivares et al., 2010). Although the Mobile Phone Problem Use Scale is one of the most frequently used instruments to assess mobile phone addiction, other instruments exist including the Mobile Phone Usability Questionnaire (MPUQ; Ryu and Smith-Jackson, 2006) and Problematic Mobile Phone Use Questionnaire (PMPUQ; Billieux et al., 2008). In Eastern countries, three scales have been developed: the Mobile Phone Dependence Inventory (MPDI; Xu et al., 2008); the Excessive Cellular Phone Use Survey (ECPUS; Ha et al., 2008), and the Smartphone Addiction Scale (SAS-SV; Kwon et al., 2013). At present there are 5 instruments translated into Spanish: the first one, already mentioned previously—MPPUS, Bianchi and Phillips (2005)—has been adapted by López-Fernández et al. (2012); the second one is the Cell-phone Over-use Scale (COS; Jenaro et al., 2007) for university populations; the third one is the Questionnaire of Mobile-Related Experiences (CERM; Fargues et al., 2009) for adult populations; the fourth one is the Test for Mobile Phone Dependence [TMD] (Chóliz, 2012) for adolescents (including a new reduced version, Chóliz et al., 2016), and finally, the fifth is a questionnaire that focuses only on the dimension of Craving (De-Sola et al., 2017b).

However, no studies have been conducted in Spain to identify mobile phone addiction in young adults using the DSM-5 criteria (American Psychiatric Association, 2012). To this end, it is necessary to develop a valid and reliable instrument measuring mobile addiction, having in mind the modifications made in the DSM-5 (American Psychiatric Association, 2012). These modifications imply that mobile phone addiction should be considered in relation to substance use disorders and behavioral addictions.The diagnostic symptoms of substance use disorders since recently include a new criterion, *craving*, featured in the DSM-5 (American Psychiatric Association, 2012). One of the most accepted definitions of craving is that of compulsive craving—an irrational and intense desire or uncontrollable compulsion to consume a particular psychoactive substance and/or perform a certain behavior, which leads to compulsive search rituals (Blasco et al., 2008; Igarashi et al., 2008; De-Sola et al., 2017b). Hence, craving should be considered as a criterion to establish a diagnosis and understand the different mediating variables when developing treatments, analyzing relapses, and designing prevention strategies. Therefore, the main purpose of this study was to develop and validate a questionnaire to measure mobile phone dependence among young Spanish speaking adults.

## Methods

### Participants

The sample comprised of community-dwelling young adults between 17 and 45 years of age (mean 21.12 years old, standard deviation = 3.05, 62.38% women and 37.62% men). They were recruited from the student population of the University of Granada. Participants were recruited by university faculty during class breaks and were selected using a probabilistic sampling design. In particular, a cluster stratified sample design was adopted. Strata were based on the different university faculties. Cluster samples were extracted such that majors and years of study were represented in proportion to the total number of students in each faculty. Finally, all students of the cluster sample were included in the final sample. There were 856 participants recruited between September 2013 and June 2014. The participants were informed about the aims of the study and provided signed informed consent prior to participation. Inclusion criteria were having a mobile phone, wanting to participate, and signing the informed consent form. Prior to recruitment the study was approved by the Research Ethics Committee from the University of Granada, Spain.

### Measures

#### The mobile phone abuse questionnaire (ATeMo)

The Mobile Phone Abuse Questionnaire (ATeMo) was developed to assess mobile phone dependence. It consists of 25 items covering addictive symptoms, based on the diagnostic criteria for behavioral addiction (gambling) and the DSM-5 (American Psychiatric Association, 2012), and also taking into account substance abuse disorders, and instruments that measure addiction to mobile phones, internet, and social networks. The addictive symptoms considered were craving, loss of control, negative life consequences, and withdrawal syndrome. Specifically, the questionnaire assessed the use of the mobile phone, the disturbance of daily activities, the increase in time spent to obtain the same satisfaction, loss of control, difficulties in stopping using the phone and the irritability produced, and the negative feelings experienced when the mobile phone cannot be used. The 25 items were answered on a 5-point Likert scale that ranged from 0 (strongly disagree) to 4 (strongly agree), resulting in a final score between 0 and 100 (see Table 1).

**Table 1:** Mobile Phone Abuse Questionnaire (ATeMo).

1. When I forget my mobile phone I feel restless.
(*Cuando me olvido del móvil me siento intranquilo*)
2. I'd rather lose my wallet than my mobile phone.
(*Prefiero perder la cartera que el móvil*)
3. I don't want to go to places where the mobile signal is weak.
(*No quiero ir a lugares donde la señal de móvil sea débil*)
4. When I travel I often touch my mobile phone.
(*Cuando viajo suelo estar tocando el móvil*)
5. I use whatsapp/line or similar more than 4 hours a day.
(*Uso whatsapp/line o similar más de 4 horas al día*)
6. I use whatsapp/line or similar while I study/work.
(*Uso whatsapp/line o similar mientras estudio/trabajo*)
7. I use whatsapp/line or similar when I'm with my friends or my family.
(*Uso whatsapp/line o similar cuando estoy con amigos o familia*)
8. I use whatsapp/line or similar at night, in bed before going to sleep.
(*Uso whatsapp/line o similar por la noche, en la cama antes de dormir*)
9. I unconsciously check whatsapp or the messages I have.
(*Inconscientemente compruebo los whatsapp o mensajes que tengo*)
10. I feel happy when I receive a message or whatsapp.
(*Me siento feliz cuando recibo un mensaje o whatsapp*)
11. I express my feelings better through whatsapp than talking.
(*Expreso mis sentimientos mejor con los whatsapp que hablando*)
12. I can never spend enough time on my mobile phone.
(*Nunca puedo estar el tiempo que necesito utilizando mi móvil*)
13. I have used my mobile phone to make myself feel better when I was feeling down.
(*Utilizo el móvil para sentirme mejor cuando estoy bajo de ánimo*)
14. I lose sleep due to the time I spend on my mobile phone.
(*Pierdo horas de sueño utilizando el móvil*)
15. When out of range for some time, I become preoccupied about the thought of missing a whatsapp or message.
(*Cuando estoy sin cobertura me preocupo por los whatsapp o mensajes que pueda perder*)
16. I have attempted to spend less time on my mobile phone but I'm unable to.
(*He intentado gastar menos tiempo usando el móvil pero no lo consigo*)
17. I have aches and pains that are associated with my mobile phone use.
(*Tengo molestias físicas o dolores producidos por el uso del móvil*)
18. I become irritable if I have to switch off my mobile phone for a meeting, dinner engagements or when at the movies.
(*Me irrita tener que apagar el móvil en situaciones como reuniones, cenas, encuentros o en el cine*)
19. I feel lost without my mobile phone.
(*Me siento perdido sin mi teléfono móvil*)
20. I feel angry if someone interrupts me when I'm using my mobile phone.
(*Me enfado si alguien me interrumpe cuando estoy usando móvil*)
21. If I had to spend 6 h without using my cell phone, I would feel restless or nervous.
(*Si tuviera que pasar 6 horas sin utilizar el móvil, me sentiría inquieto o nervioso*)
22. I feel bored when I'm not using my mobile phone.
(*Me siento aburrido cuando no estoy utilizando el móvil*)
23. I neglect my work or class assignments to use my mobile phone.
(*Desatiendo mis tareas de trabajo o clase para usar el móvil*)
24. I ignore my friends to use my mobile phone.
(*Desatiendo a mis amigos para usar el móvil*)
25. I ignore my family to use my mobile phone.
(*Desatiendo a mi familia para usar el móvil*)

Instructions: We are interested in how people use mobile phones to communicate. Please indicate the degree to which you agree or disagree with each of the following statements regarding your use of your mobile phone on the following scale:

*0, strongly disagree; 1, disagree; 2, neutral; 3, agree; and 4, strongly agree*.

*Scoring and interpretation: Sum the items in parenthesis for subscale scoring: Craving (1, 2, 3, 4, 9, 10, 12, 16), Loss of Control (5, 8, 13, 14), Negative Life Consequences (6, 7, 11, 17, 18, 23, 24, 25) and Withdrawal Syndrome (15, 19, 20, 21, 22)*.

#### The MULTICAGE CAD-4

The MULTICAGE CAD-4 was designed to screen for a history of drug abuse and addiction behavior. It assesses alcoholism (items 1–4), gambling disorders (items 5–8), drug addiction (items 9–12), eating disorders (items 13–16), internet addiction (items 17–20), video gaming addiction (items 21–24), compulsive buying disorder (items 25–28) and sex addiction (items 29–32). The psychometric properties have been well established in Spanish adult populations. It demonstrates high > 0.7 Cronbach's alpha coefficient. In the exploratory factor analysis, 8 components are identified that identify the proposed structure the diagnostic sensitivity for alcohol was 92.4%, and between 94 and 100% for heroin, cocaine and cannabis (Pedrero-Pérez et al., 2007).

### Procedure

The study consisted of two stages: in the first stage the instrument was developed and in the second stage it was validated. The construction of the Mobile Phone Abuse Questionnaire (ATeMo) was based on the DSM-5 (American Psychiatric Association, 2012) that does not recognize mobile addiction as a disorder but makes reference to tobacco addiction and gambling. Ideas were taken from instruments that measure addictions to mobile phones, internet, and social networks and items were created taking into account all the aforementioned. For the construction of the items, criteria for constructing items for Likert questionnaires were used (Jenaro et al., 2007; Billieux et al., 2008; Fargues et al., 2009; Chóliz, 2012; Chóliz et al., 2016; De-Sola et al., 2017a). This set of defined criteria together with the items that evaluated them were reviewed by three experts on clinical psychology, educational psychology, and psychometrics. The experts collaborated in writing and ensuring the understanding, clarity, and consistency in the definitions of the criteria and the items. For the evaluation of the items a5-point rating system was applied (from 0 to 4) taking into account the frequency from never to always (Fishman and Galguera, 2003; Schepers, 2009; Furr, 2011; DeVellis, 2012). Once the expert evaluation was concluded, a pilot experiment was carried out on a sample of 65 university students. They were asked to indicate whether the items in the questionnaire were comprehensible or not, encouraging them to raise any doubts that they had regarding each item.

The instrument was then administered to the final sample of participants in order to establish its validity. The data were collected from students of the University of Granada through stratified sampling by conglomerates, according to majors and groups of the different degrees taught at the University of Granada (Psychology, Speech Therapy, Tourism, English, History, Literature, GADE, Economy, Biology, Physics, Optics, Primary, Infant, Pedagogy, Law, Medicine, Pharmacy, Social Work, Policies, Sociology, Information Technology, Roads and Telecommunications). Teachers responsible for the selected groups were sent an email informing them of the objectives of the study and requesting their help so that the students could participate. They were asked to inform their students about the study and the time during breaks was used to complete the questionnaires. It was emphasized that the participation was voluntary, that is, the students were free not to participate if they preferred it.The teachers also emphasized the need for honesty when filling out the survey and guaranteed the confidentiality of the responses. The survey started with short demographic questions (sex and age) followed by the ATeMo questionnaire.

### Data analysis

To obtain empirical evidence about the construct validity of the questionnaire and given the ordinal nature of the data, we conducted Confirmatory Factor Analysis (CFA) using polychoric correlations and Unweighted Least Squares (ULS) as estimation method (Hernández et al., 2000; Yang-Wallentin et al., 2010; Morata-Ramírez et al., 2015). We also tested the basic psychometric properties of the dimensions obtained (mean, standard deviation, reliability and discrimination). For criterion validity, correlation analysis was performed to determine the relationship of the ATeMo factors and the sub-dimensions of the MULTICAGE CAD-4. Gender differences between the different factors of the questionnaire were also examined through a MANOVA. Finally, to achieve an initial approximate interpretation of the scores, we calculated the percentiles in the total sample and split them by gender. The statistical programs used were SPSS 15.0 for Windows and LISREL 8.71 (Jöreskog and Sörbom, 1996).

## Results

### Confirmatory factor analysis (CFA)

**Figure 1:**
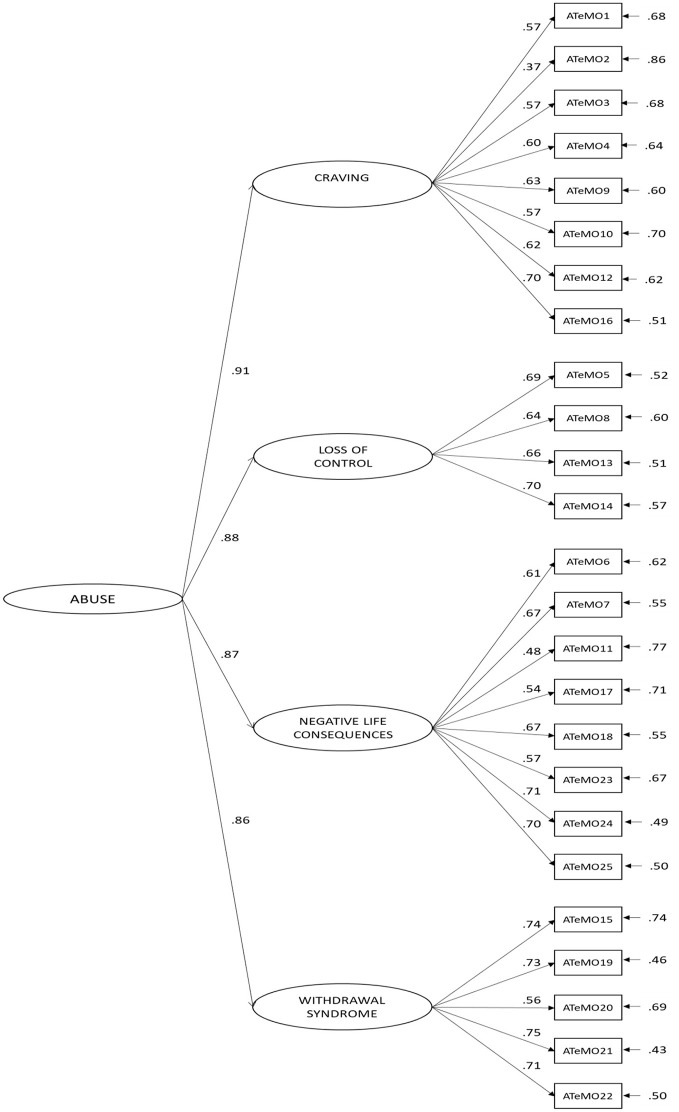
χ2 = 274.18; d.f. = 265; *p* = 0.34; RMSEA = 0.021, GFI = 0.97; AGFI = 0.97; CFI = 1; NFI = 1; and SRMR = 0.06.

In order to obtain empirical evidence about the adequateness of the postulated structure ofthe ATeMo questionnaire, a CFA was conducted. In line with the theoretical background, the dimensional structure considered implied a general second-order factor referring to mobile phone dependence and four first order factors. The four first order factors were the following: eight items contributed to the first factor of Craving (1, 2, 3, 4, 9, 10, 12, 16), four items to the second factor of Loss of Control (5, 8, 13, 14), eight items to the third factor of Negative Life Consequences (6, 7, 11, 17, 18, 23, 24, 25) and five items to the fourth factor Withdrawal Syndrome (15, 19, 20, 21, 22). For the model examined (Figure 1), the Global fit Indices were: χ2 = 274.18; *d.f*. = 265; *p* = 0.34. The value of the Root Mean Square Error of Approximation (*RMSEA*) was 0.021, with a 90% interval between 0.0 and 0.050. The Goodness of Fit Index (*GFI*) was 0.97, the Adjusted Goodness of Fit Index (*AGFI*) was 0.97, the comparative Fit Index (*CFI*) was 1, the Normed Fit Index (*NFI*) was 1 and the Standardized Root Mean Square Residual (*SRMR*) was 0.06. These data show that the fit values of the model are appropriate. All the lambdas and gammas parameters were statistically significant.

### Reliability

The reliability of ATeMo was assessed using Cronbach's alpha coefficients (**Table 3**) and the resulting values were: Total score 0.91; Craving factor 0.74; the Loss of Control factor 0.70; Negative Life Consequences factor 0.77; and for the Withdrawal Syndrome factor 0.77. In addition, we calculated descriptors for the ATeMo from the CFA (mean, standard deviation and mean discrimination of the items of each dimension: Table 2).

**Table 2:** Cronbach's alpha coefficients and the mean, standard deviation, and mean discrimination for the AteMo questionnaire derived from the CFA.

	**F1 (Cv)**	**F2 (LC)**	**F3 (NLC)**	**F4 (WS)**	**ATeMo (TS)**
Mean	11.85	6.71	7.48	4.02	30.07
Standard deviation	5.39	3.66	4.72	3.53	15.00
Cronbach's α	0.74	0.70	0.77	0.77	0.91
Mean discrimination	0.45	0.49	0.48	0.55	0.52

*Cv, Craving; LC, Loss of Control; NLC, Negative Life Consequences; WS, Withdrawal Syndrome; and TS, Total Scores*.

### Criterion validity

To determine the criterion validity, we calculated the Pearson bivariate correlation index between the total score and each of the ATeMo factors, as well as with the MULTICAGE CAD-4 subscales (see Table 3). There was a positive correlation between the ATeMo total score and Alcoholism, Gambling disorders, Internet addiction, and Compulsive buying in the MULTICAGE CAD-4 subscales. Furthermore, there was a positive correlation between the Negative Life Consequences factor of ATeMo and Drug addiction in the MULTICAGE CAD-4 subscale; the Craving and Loss of Control ATeMo factors and Video game addiction in the MULTICAGE CAD-4 subscale; and the Negative Life Consequences and Withdrawal Syndrome factors of ATeMo and Sex addiction in the MULTICAGE CAD-4 subscale.

**Table 3:** Correlations between the total score, the factors of ATeMo, and the MULTICAGE CAD-4 subscales.

	**MULTICAGE CAD-4**							
	**1**	**2**	**3**	**4**	**5**	**6**	**7**	**8**
F1(Cv)	0.127^**^	−0.001	0.013	0.210^**^	0.252^**^	−0.073^*^	0.287^**^	0.032
F2 (LC)	0.139^**^	−0.036	0.025	0.193^**^	0.218^**^	−0.075^*^	0.260^**^	012
F3(NLC)	0.196^**^	0.062	0.100^*^	0.172^**^	0.300^**^	0.037	0.305^**^	093^**^
F4 (WS)	0.163^**^	0.022	0.032	0.161^**^	0.293^**^	−0.047	0.278^**^	0.073^*^
ATeMO (TS)	0.180^**^	0.015	0.050	0.214^**^	0.307^**^	−0.044	0.328^**^	0.061

*1, Alcoholism; 2, Gambling disorders; 3, Drug addiction; 4, Eating disorders; 5, Internet addiction; 6, Video games addiction; 7, Compulsive buying; 8, Sex addiction; F (Cv), Craving; F2 (LC), Loss of Control; F3 (NLC), Negative Life Consequences; F4 (WS), Withdrawal Syndrome; and ATeMO (TS), Total Scores*.

* *p < 0.05*,

** *p < 0.01*,

***p < 0.001.

In general terms, the direction of the correlations is consistent with what was expected, however, given the large sample size, correlations of 0.073 (Craving-Video games addiction), for example, result statistically significant. For this reason, according to Rosnow and Rosenthal (1996) none of the correlations presents a large effect size (|*r*| > 0.37). The effect size is medium (|*r*| > 0.24) for the correlations between the factors of ATeMO with Internet Addiction, and Compulsive Buying. The correlation between the factors of ATeMO and Alcoholism and Eating Disorders have a low effect size (|*r*| > 0.10). All other correlations have an irrelevant effect size.

### Score interpretation

In order to provide preliminary data to help interpret the scores obtained, the 10th to 90th percentiles are presented for the total sample, and for men and women separately (Table 4).

**Table 4:** Percentiles, raw scores in ATeMo, mean and standard deviation for men and women.

**Pc**	**Raw Scores**		
	**Men**	**Woman**	**Total**
10	1	2	2
20	2	4	3
30	3	5	5
40	4	7	6
50	5	8	7
60	6	9	8
70	7	10	9
80	8	11	10
90	10	12	12
Mean	5.37	7.51	6.71
Sd.	3.46	3.54	3.66

*Pc, percentile; Sd, standard deviation*.

## Discussion

In the present study we have developed a new valid and reliable scale to measure mobile phone abuse and dependence in Spain (ATeMo). The ATeMo Questionnaire consists of 25 items covering addictive symptoms, based on the diagnostic criteria of the DSM-5 (American Psychiatric Association, 2012). It is evaluated on a 5-point Likert-type scale ranging from 0 (strongly disagree) to 4 (agree), resulting in a final score in the range of 0–100. According to results from a confirmatory factor analysis, the ATeMo represents a general second order factor and four first order factors consistent with addiction theory: Craving, Loss of Control, Negative Life Consequences, and Withdrawal Syndrome. These factors show considerable overlap with the symptoms proposed previously (Bianchi and Phillips, 2005; Rutland et al., 2007; Igarashi et al., 2008; Yen et al., 2009; Walsh et al., 2010; Chóliz, 2012; Merlo et al., 2013; Chóliz et al., 2016) and were developed according to the criteria for the diagnostic symptoms of substance dependence disorders in the DSM-IV-TR (American Psychiatric Association, 2002) and the DSM-5 (American Psychiatric Association, 2012), the latter more recently including craving as a diagnostic criterion.

In assessing the reliability of the ATeMo questionnaire, Cronbach's alpha coefficients were calculated, demonstrating it had excellent internal consistency as seen elsewhere in similar studies in Spain (Chóliz, 2012; López-Fernández et al., 2012; Vanyukov et al., 2012; Chóliz et al., 2016). These coefficients were higher than those obtained in some previous studies (Fargues et al., 2009), where measures were developed according to the criteria for diagnosing symptoms of substance dependence disorders in DSM-IV-TR (American Psychiatric Association, 2002). The MULTICAGE CAD-4 subscales were used to determine potential criterion validity of ATeMo, identifying a positive correlation between the ATeMo total score and Alcoholism, Drug addiction, Eating disorders, Internet addiction, and Compulsive Buying subscales (Chiu et al., 2013; Gallimberti et al., 2016; Jiang and Shi, 2016; De-Sola et al., 2017a). Furthermore, there was a positive correlation between the Craving ATeMo factor, Alcoholism, Eating disorders, and Internet addiction, and a negative correlation with Video gaming addiction in the MULTICAGE CAD-4 subscale. Similarly, there was a positive correlation between the ATeMo factor Loss of Control and Alcoholism, Eating disorders, Internet addiction, and Compulsive buying, as well as a negative correlation with Gambling Disorders in the MULTICAGE CAD-4 subscale. This is consistent with the positive correlation between self-control and addiction identified previously (Jiang and Shi, 2016). Again, there was a positive correlation with Negative Life Consequences as an ATeMo factor and Alcoholism, Drug addiction, Eating disorders, Internet addiction, Compulsive buying, and Sex addiction in the MULTICAGE CAD-4 subscale, and there was a similarly positive correlation between Withdrawal Syndrome as an ATeMo factor and Alcoholism, Eating disorder, Internet addiction, Video gaming addiction, and Compulsive buying. Indeed, loss of control, negative life consequences and withdrawal syndrome were already considered as diagnostic criteria for addiction disorders prior to DSM-5 (American Psychiatric Association, 2012).

The relationships described above are consistent with previous considerations that alcohol consumption may predict problematic mobile phone use (De-Sola et al., 2017a). They are also consistent with previous results on the relationship between Internet and mobile phone addiction (Chiu et al., 2013) and with previous results suggesting common impulsive aspects between compulsive buying and mobile phone addiction (Jiang and Shi, 2016).

Furthermore, the survey conducted indicated a common continuum of substance abuse and behavioral addictions, as identified previously in surveys that focused on such co-morbidity (Chiu et al., 2013; Jiang and Shi, 2016; De-Sola et al., 2017a; although an association between eating disorders and mobile phone abuse is yet to be found). These results suggest that alcohol, drugs, and pathological gambling may not be the only crippling addictions. Addiction statistics are scarce because many destructive habits (such as gaming, shopping, sex, etc.) are not yet officially recognized as addictions, although they could be problematic for many reasons. Some of these involve the direct manipulation of pleasure through the consumption of products like in the case of drug use disorders and food-related disorders.

The results obtained with ATeMo indicate that there are gender differences between males and females regarding mobile phone abuse, with scores ≥8 for the former and ≥10 for the latter potentially indicating mobile phone addiction. These results are consistent with previous findings indicating that females send more and longer texts, they talk for longer than men on the phone, and tend to regard mobile phones as a social tool (Roberts et al., 2014).

Our findings demonstrate that the ATeMo is a valid and reliable instrument that can be administered to different groups of university students. In addition, while this instrument was developed for university students, renewed construct validity and reliability analyses could convert it into a version suitable for adolescents.

Our results should be evaluated in view of several important limitations. First, the sample used in this study was relatively homogeneous with respect to age and educational level. Second, mobile phone addiction should be investigated in relation to a number of variables, such as demographic, personality, and clinical characteristics. This could advance our understanding of the interaction of humans with technology, as well as our understanding of the nature and causes of technology-related addictions. Overall, taking into account the lack of a valid and reliable questionnaire to measure the addiction to the mobile phone, ATeMo could be an adequate instrument to measure the mobile phone addiction in future investigations.

Regarding clinical implications, the development of the ATeMo questionnaire to detect mobile phone abuse is an important step in the development of diagnostic and treatment procedures and in the design of prevention and intervention strategies.

In future studies, it would be of interest to examine the problems associated with mobile phone use in relation to variables such as solitude, depression, self-esteem, well-being, academic success, and other demographic variables. Further studies into the problematic use of mobile phones will not only allow us to better understand this problem but they should provide information to aid the committees determining future DSM criteria, especially in relation to addictions associated with new technologies. Moreover, a more profound analysis thorough ROC curves of the cut-off thresholds should be performed to help interpret the scores obtained and to classify the subjects. Moreover, other construct validity evidences should be investigated. In this sense, invariance analysis by gender, of age group, for example, is necessary to obtain empirical evidences about the equivalence in the constructs and items operatized in ATeMO. Once guarantee this issue, Differential Item Functioning and a deep comparative analysis by the sorting variables considered will be necessary to ensure that the decisions made based on the test scores are valid.

In summary, we have developed a scale to measure Mobile Phone Abuse, ATeMo, that takes into account the criteria for the diagnosis of substance use or addiction described in DSM-5 (American Psychiatric Association, 2002). The evaluation of craving was an important aspect of this questionnaire, as previously no measures existed that were consistent with the DSM-5 (American Psychiatric Association, 2012) criteria. The majority of measures had been developed based on the literature on substance use and addiction (Toda et al., 2004; Bianchi and Phillips, 2005; Rutland et al., 2007; Igarashi et al., 2008; Yen et al., 2009; Walsh et al., 2010; Chóliz, 2012; López-Fernández et al., 2012; Merlo et al., 2013; Chóliz et al., 2016), and the items in most of the previous instruments reflect the diagnostic criteria for substance use or addiction described in DSM-IV-TR (American Psychiatric Association, 2012). Based on the current findings we can conclude that the ATeMo questionnaire has satisfactory reliability and validity, having included craving as a diagnostic criteria for dependence.

## Availability of data and materials

R code and data are available from the authors under request.

## Ethics statement

This study was approved by the Research Ethics Committee from the Granada University. All procedures performed in our study involving human participants were in accordance with the ethical standards of the institutional research committee and with the 1964 Helsinki declaration and its later amendments or comparable ethical standards. Informed consent was obtained from all individual participants included in the study.

## Author contributions

All the authors participated in the conception and design of the work, specifically MO-C and FL-T, conceived the original idea for the study, obtained funding and wrote the study protocol. MO-C manages the day to day running of the study, including all participant follow-up and IR-U and PH-T undertaked all data analyses. This study paper was written by FL-T, IR-U, and PH-T with input from all co-authors. All authors read and approved the final manuscript and believe that the manuscript represents valid work; carefully read and fully approve of it.

### Conflict of interest statement

The authors declare that the research was conducted in the absence of any commercial or financial relationships that could be construed as a potential conflict of interest.
